# The “Ins and Outs and What-Abouts” of H2A.Z: A tribute to C. David Allis

**DOI:** 10.1016/j.jbc.2025.108154

**Published:** 2025-01-04

**Authors:** Felix Diegmüller, Jörg Leers, Sandra B. Hake

**Affiliations:** Institute for Genetics, Justus-Liebig-University Giessen, Giessen, Germany

**Keywords:** C. David Allis, H2A.Z, chaperones, gene regulation, development, differentiation, cancer, oncohistones

## Abstract

In 2023, the brilliant chromatin biologist C. David Allis passed away leaving a large void in the scientific community and broken hearts in his family and friends. With this review, we want to tribute Dave’s enduring inspiration by focusing on the histone variant H2A.Z, a nucleosome component he was the first to discover as *hv1* in *Tetrahymena*. We summarize the latest findings from the past 5 years regarding the mammalian H2A.Z histone, focusing on its deposition and eviction mechanisms, its roles in transcriptional regulation, DNA damage repair, chromatin structure organization, and embryonic development, as well as how its deregulation or mutation(s) of its histone chaperones contribute to disease development. As Dave liked to say ‘*Every amino acid matters’*; the discovery and characterization of functionally different H2A.Z’s isoforms, which vary only in three amino acids, prove him—once again—right.

## C. David Allis: “*Every amino acid matters*”—Spot-on characterization of histone variants

In January 2023, C. David Allis, or “Dave” to his colleagues and friends, unexpectedly passed away, leaving a large void in the scientific community and broken hearts in his family and friends. Numerous obituaries have honored his remarkable life, generosity, and kindness, as well as his legacy as an inspiring scientist and dedicated mentor (1, 2, 3). These tributes offer a glimpse of his groundbreaking discoveries and his uniquely cheerful character.

Among his many achievements, Dave fundamentally altered our understanding of the packaging structure of DNA. He and several other researchers revealed the enzymatic toolkit that regulates posttranslational modifications (PTMs) of histones, which in turn control DNA-related processes. His work focused on nucleosomes, which make up the chromatin structure with their four core histone proteins H2A, H2B, H3, and H4. Together with Brian Strahl, he proposed the famous “Histone Code Hypothesis” in 2000 (4), which has since paved the way for countless new discoveries in chromatin biology worldwide. One of Dave’s well-known and memorable statements to his research group and at conferences was “Every amino acid matters” quickly followed by “…but people matter more” (citation: C. David Allis). The first statement aptly reflects the chemical nature of histones and, particularly histone variants.

Before his groundbreaking work on histone PTMs, one of Dave’s earliest discoveries in the chromatin field was the identification of two types of histone H3 proteins (variants) in the ciliate *Tetrahymena thermophila* in 1979 (5). He demonstrated that these histone variants play a crucial role in the structural and functional differentiation of chromatin (6). Additionally, he showed that other variants, such as H2A.Z (originally named *hv1* by Dave) are conserved across species and are also present in mammals (7).

These intriguing proteins differ from their “canonical” replication dependently expressed counterparts in their amino acid sequences, sometimes differing at only few sites, and convey specialized functions. They are expressed throughout the cell cycle and are deposited into and evicted from chromatin by specialized chaperones and/or remodeling complexes (8). Once integrated into chromatin, histone variants can alter nucleosome stability, enable distinct PTM patterns, and facilitate the recruitment of specific proteins (9). Since the initial discovery of histone variants in lower eukaryotes, the number and diversity of these—sometimes essential—proteins identified across all eukaryotic clades have increased substantially.

In this review, dedicated to Dave in recognition of his enduring inspiration, we focus on the histone variant H2A.Z (“Dave’s” hv1) in mammals. We will summarize the latest findings from the past 5 years concerning its deposition and eviction mechanisms, roles in transcriptional regulation, DNA damage repair, chromatin structure organization, and embryonic development. Additionally, we will discuss how its deregulation and/or mutation(s) of its histone chaperones contribute to disease development.

## H2A.Z: what do we know so far?—Introducing an enigmatic histone variant

In this review, we focus on H2A.Z because it is one of the most intriguing and enigmatic histone variants, widely studied across laboratories worldwide. Despite sharing only 60% identity with the replication-dependent H2A, H2A.Z has unique functional properties. H2A.Z plays a key role in nearly all DNA-related mechanisms, including transcriptional activation and repression, DNA damage recognition and repair, nucleosome stability, chromosome organization as well as stem cell differentiation and maintenance (10, 11). It is an evolutionarily conserved histone variant found in all eukaryotes studied so far (12) and is essential for survival in most higher organisms (13).

**Figure 1:**
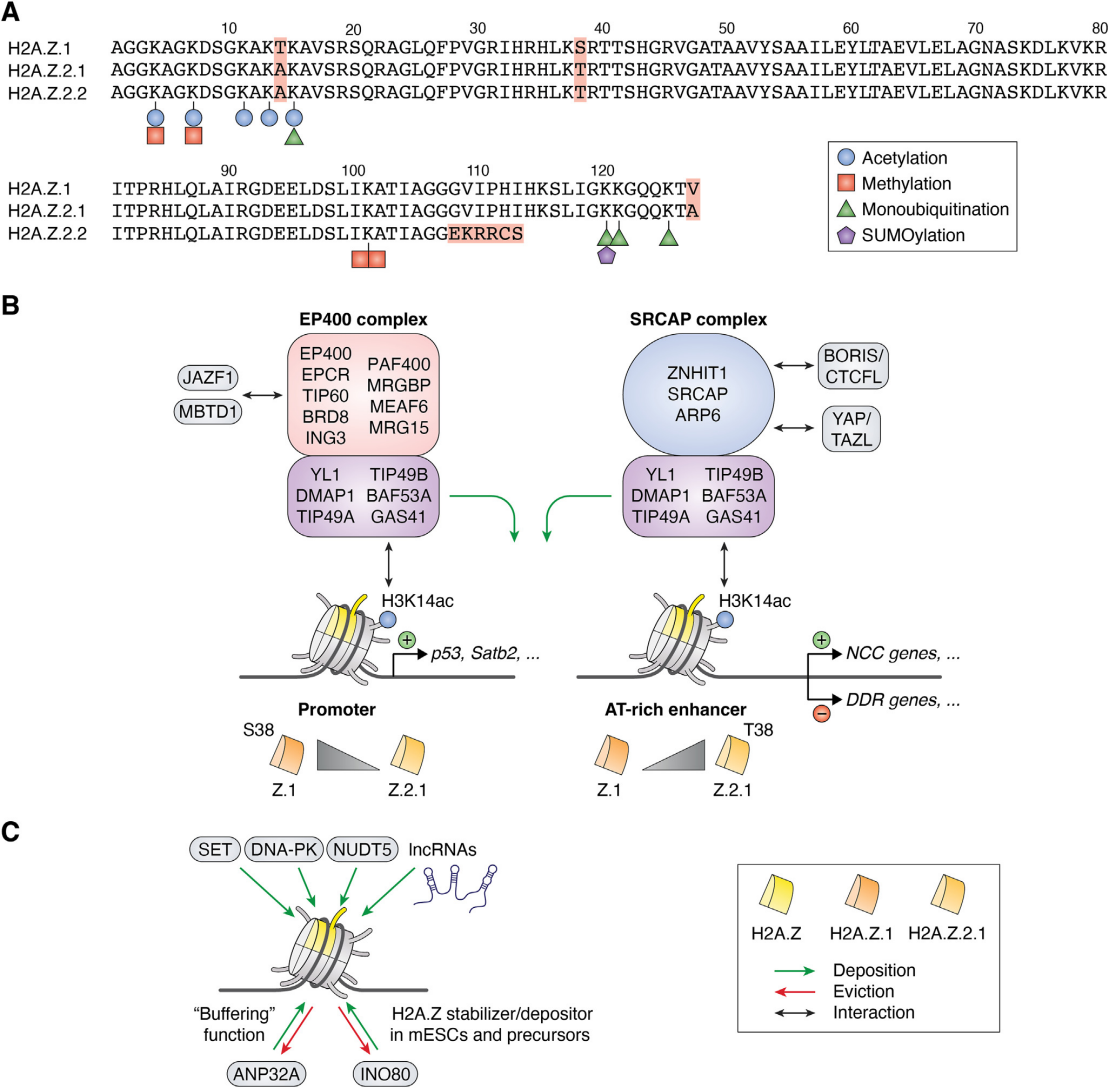
**Human H2A.Z isoforms and their intricate “In and Out” mediating complexes**. *A*, “Every amino acid matters”: small amino acid differences in H2A.Z isoforms. Alignment of human H2A.Z.1, H2A.Z.2.1, and H2A.Z.2.2 protein sequences. Identical amino acids are displayed on a *white background*, while amino acid variations among the H2A.Z isoforms are highlighted in *red*. PTMs are indicated as follows: acetylation sites are marked as *blue circles*, methylation as *red squares* (with one square indicating monomethylation and two squares indicating dimethylation), monoubiquitination as *green triangles* and SUMOylation as *purple pentagons*. *B*, deposition of H2A.Z by EP400 and SRCAP complexes. Depositors of H2A.Z and interacting proteins are illustrated as described in the text. H2A.Z.1 is depicted in *dark orange*, H2A.Z.2.1 in *light orange*, unspecified H2A.Z isoform is shown in *yellow*. H2A.Z.1 is preferentially incorporated in promoter regions and has a serine at position 38; H2A.Z.2.1 is enriched at AT-rich enhancers and has a threonine at position 38. Gene activation is represented by *green* “+”, and gene silencing is indicated by *red* “-” *circles*. Acetylation = *blue circles*. *C*, H2A.Z’s novel depositors and well-known evictors with unusual additional functions. SET, DNA-PK, NUTD5, and long noncoding RNAs (lncRNAs) have been identified to (indirectly) mediate H2A.Z chromatin deposition (*green arrow*).The well-known H2A.Z evictors (*red arrow*) ANP32E and INO80 show additional buffering and stabilization functions. PTM, posttranslational modifications; ANP32E, acidic nuclear. phosphoprotein 32 family member E; SRCAP, snf2-related CREBBP; DNA-PK, DNA-dependent protein kinase.

In vertebrates, two distinct genes, *H2AFZ* and *H2AFV*, encode two highly similar isoform proteins, H2A.Z.1 and H2A.Z.2(0.1), differing by just three amino acids (14) (Fig. 1*A*). Restricted to primates, alternative splicing of the *H2AFV* gene produces an isoform called H2A.Z.2.2, which has a shortened and unique C terminus. This alteration leads to destabilization of the nucleosome structure when H2A.Z.2.2 is incorporated into chromatin (15, 16).

For a long time, the distinct functions of the two major H2A.Z isoforms, H2A.Z.1 and H2A.Z.2.1, remained uncertain, as both are found at similar genomic locations and seem to use the same deposition machineries. The first indication of functional differences between H2A.Z.1 and H2A.Z.2.1 emerged when it was discovered that the acetyl-lysine reader bromodomain containing protein 2 (BRD2) preferentially binds to H2A.Z.1 nucleosomes over those containing H2A.Z.2.1 (17). However, distinguishing between these two highly similar histone variants remains challenging due to the absence of antibodies that can reliably differentiate between them. As a result, many studies rely on overexpressed and tagged exogenous versions of these variant proteins. This necessitates cautious interpretation, since altered expression levels or the presence of a tag may affect their function(s). Nonetheless, recent depletion studies in rat cortical neurons and cell lines targeting *H2AFZ* or *H2AFV* have provided evidence that these variants have distinct roles, such as in neuronal gene regulation (18) and cell division control (19).

H2A.Z is typically found within nucleosomes surrounding regulatory regions, such as promoters and enhancers (20, 21), as well as in pericentric heterochromatin (PCH) (22). Additionally, it is often removed from regions with DNA-damage (23). Not surprisingly, H2A.Z is primarily involved in the regulation of gene transcription, exerting both activating and repressive effects depending on the genomic context (10). This dual role as activator and silencer of gene expression may be attributed to its various PTMs, including acetylation, methylation, ubiquitination and SUMOylation (Fig. 1*A*). For instance, hyperacetylation of five lysines (Ks) within H2A.Z’s N-terminus is associated with gene activation (24), while monomethylation of H2A.ZK7 is required for mouse embryonic stem cell (mESC) self-renewal (25). SUMOylation of H2A.Z.2.1 regulates its exchange at DNA damage sites (26), and monoubiquitination of H2A.Z’s C-terminal lysines is found in heterochromatin and linked to transcriptional repression (27, 28). Additionally, H2A.Z plays a critical role in (neuro-) development (29, 30) and in responses to environmental changes (31). It is not surprising that dysregulation of H2A.Z itself or its associated proteins—depositors, evictors, writers, erasers, and readers—are implicated in various diseases.

All H2A.Z isoforms are deposited into chromatin by two large, multisubunit remodeler complexes: the p400/TIP60/NuA4 (EP400) and snf2-related CREBBP (SRCAP) complexes (Fig. 1*B*), both of which evolved from the SWI2/SNF2-related 1 (SWR1) complex found in lower eukaryotes (32). The removal of H2A.Z variants from chromatin is facilitated by the acidic nuclear phosphoprotein 32 family member E (ANP32E) (33, 34) and inositol-requiring mutant 80 (INO80) (35, 36). These chaperones, remodelers and evictors govern the precise genome-wide distribution of H2A.Z through targeting mechanisms that remain poorly understood.

## Recent discoveries of the “Ins and Outs” of H2A.Z

### How does SRCAP deposit H2A.Z.1 and H2A.Z.2?

In the past 5 years, significant advances have been made in understanding the unique chromatin depositors and evictors of H2A.Z (Fig. 1, *B* and *C*).

Notably, the group of Joanna Wysocka discovered that mutations within the SRCAP gene, which cause the developmental disorder Floating-Harbor syndrome (FHS), lead to a loss of SRCAP nuclear localization and in turn an altered neural crest cell differentiation program resulting in craniofacial defects (37). Their findings indicate that while the genomic incorporation patterns of H2A.Z.1 and H2A.Z.2.1 are qualitatively similar, there is a genome-wide localization bias: H2A.Z.1 is more frequently localized to promoters, whereas H2A.Z.2.1 is more enriched at AT-rich enhancers (Fig. 1*B*). Since FHS-causing mutations in *SRCAP* result in a preferential loss of H2A.Z.2.1 at AT-rich enhancers with a corresponding downregulation of associated genes, it is now becoming clear that the H2A.Z isoforms are somehow differentially regulated by their two known chromatin deposition complexes. How this quantitative difference is mechanistically achieved, is still unknown to date. However, there is one intriguing clue: H2A.Z isoform specificities appear to depend on a unique residue at position 38: serine in H2A.Z.1 and threonine in H2A.Z.2.1 (37). As Dave always said: “Every amino acid matters”!

In addition to FHS, other mutations in the *SRCAP* gene have been linked to various illnesses, including clonal hematopoiesis (CH) (38), a somatic condition characterized by an expansion of hematopoietic stem cells (HSCs) in elderly individuals (39). Similar to FHS, CH-associated mutations in *SRCAP* lead to a reduction of H2A.Z at AT-rich DNA-sequences, likely due to the involvement of SRCAP’s AT-hooks in its C terminus (38). The study did not determine whether H2A.Z.2.1 localization was more affected than H2A.Z.1, as isoform-specific antibodies are lacking. In CH-associated (HSCs, the reduction of H2A.Z deposition is particularly evident at promoters of genes involved in DNA damage repair , leading to their upregulation (Fig. 1*B*). Thus, in this particular disease, H2A.Z functions as a repressor and not as an activator of transcription, highlighting again the enigmatic behavior of this variant histone.

*SRCAP* mutations have also been linked with autism spectrum disorders (40). Recent research demonstrates that *SRCAP* haploinsufficiency in mice results in autistic-like behaviors, likely due to a decreased expression of the special AT-rich sequence-binding protein 2 (Satb2) (41). This reduction is associated with deregulated H2A.Z deposition in the *Satb2* promoter.

However, these data have to be interpreted with caution. A preprint from David Sutor’s group, utilizing an innovative rapid SRCAP depletion method along with an ATPase catalytically-dead SRCAP mutant, revealed that SRCAP also possesses an H2A.Z-independent function (42). Specifically, it prevents certain transcription factors (TFs) from binding to regulatory DNA elements. This finding must be considered when examining the phenotypes of *SRCAP* mutants and deletions in other studies.

### How does SRCAP find its target sequences?

While SRCAP’s AT-hooks may facilitate binding to AT-rich regions, a recent study indicates that the nuclear protein brother of regulator of imprinted sites/CCCTC-binding factor-like (BORIS/CTCFL) recruits SRCAP to CCCTC-binding factor (CTCF) binding sites (43) (Fig. 1*B*). This recruitment activates the transcription of testis-specific genes in cancer cells by promoting H2A.Z deposition and chromatin relaxation.

Additionally, Yes-associated protein/transcriptional coactivator with PDZ-binding motif (YAP/TAZ), which are transcriptional coactivator paralogs of the Hippo pathway and deregulated in various malignant tumors, have been found to interact with the SRCAP complex (44) (Fig. 1*B*). This interaction facilitates H2A.Z deposition at the promoters of their specific target genes. Consequently, genes associated with the Hippo pathway are upregulated, indicating that H2A.Z primarily functions as transcriptional activator in this particular cellular context.

Future studies should explore whether genomic regions associated with SRCAP/BORIS or SRCAP/YAP/TAZ are targets for H2A.Z.2.1 and/or H2A.Z.1. Additionally, it will be important to determine whether other yet-to-be-identified TFs mediate site-specific recruitment of chaperone complexes that facilitate H2A.Z isoform-specific deposition.

### Unique components of the SRCAP complex regulate H2A.Z deposition

In recent years, several studies elucidated the role of zinc finger HIT-type containing protein 1 (ZNHIT1), a unique subunit of the SRCAP complex. Mechanistically, Znhit1 enhances the interaction between H2A.Z and YL1/VPS72, a member of both EP400 and SRCAP complexes, by promoting the phosphorylation of YL1 (45). Functionally, Znhit1 is essential for mediating global H2A.Z deposition in a model of intestinal epithelial crypts (45). ZNHIT also plays an important role for maintaining H2AZ occupancy and pulling H2A-H2B dimer out of the nucleosome (46).

Furthermore, Znhit1 plays a crucial role in embryonic lung morphogenesis by regulating H2A.Z deposition and, consequently, the expression of bone morphogenetic protein 4, a direct target of H2A.Z (47). During mammalian heart development, Znhit1 is critical for heart function (48); its depletion significantly suppresses the transcription of genes involved in regulating oxidative metabolism, which is accompanied by a reduction of H2A.Z deposition (49). Additionally, in HSCs, Znhit1 is vital for maintaining quiescence (50).

In male germ cells, Znhit1 is responsible for controlling meiotic initiation, which is the transition from mitosis to meiosis ensuring gamete formation (51). In these cells, Znhit1-mediated deposition of H2A.Z regulates the expression of meiotic genes.

It is increasingly clear that Znhit1 plays a critical role in the H2A.Z-dependent regulation of cell type-specific genes at various developmental stages.

### Shared subunits between SRCAP and EP400 complexes that influence H2A.Z deposition

A shared subunit of both EP400 and SRCAP complexes is the glioma amplified sequence 41 (GAS41/YEATS4) protein, which contains a Yaf9, ENL, AF9, Taf14, and Sas5 (YEATS) domain capable of recognizing acetylated lysines. A study by Kikuchi et al., suggests that GAS41 specifically recognizes H3 lysine 14 acetylation (H3K14ac) through its YEATS domain (Fig. 1*B*), which is also essential for the chromatin occupancy of both H2A.Z and GAS41 (52). Thus, site-specific H2A.Z deposition appears to be influenced not only by DNA sequences, such as AT-rich regions, and the SRCAP interaction partners like BORIS/CTCFL or YAP/TAZ, but also by the chromatin environment, particularly the presence of H3K14ac.

In uterine leiomyomas, which are benign smooth muscle tumors common in premenopausal women, both somatic and germline mutations of *GAS41*, *ZNHIT1*, *DMAP1*, and *ACTL6A* subunits have been identified (53, 54). These mutations lead to a decrease in H2A.Z levels. In this particular context, loss of H2A.Z leads to epigenetic instability and chromatin becomes more open, resulting in an upregulation of *e.g.* CBX family member genes (53).

Surprisingly, in addition to its role in gene regulation, H2A.Z also appears to be required for nuclear reassembly after mitosis (55). Depletion of YL1, the H2A.Z-interacting subunit of both the EP400 and SRCAP chaperone complexes, or of H2A.Z itself, results in malformed and nonfunctional nuclei, as well as cells with prolonged telophase. Interestingly, this YL1-dependent H2A.Z deposition event does not require the involvement of other members of these two chaperone complexes, as suggested by data from a cell-free assay using *Xenopus* egg extracts (55).

### Unique EP400 complex members and their functions

Conditional depletion of the *Ep400* gene, the namesake subunit of this ATP-dependent remodeler complex, in mouse Schwann cell leads to a peripheral neuropathy characterized by a substantial redistribution of H2A.Z (56). Interestingly, H2A.Z is not completely lost from chromatin; instead, its levels are reduced at certain sites while increasing at others. This observation contrasts with findings from *SRCAP* mutations or depletion, which result in a site-specific loss of H2A.Z. This discrepancy raises an important question: why are two distinct chaperone/remodeling complexes necessary for H2A.Z deposition, and what accounts for their differing mechanisms of action?

The bromodomain-containing protein 8 (BRD8), a unique subunit of the EP400 complex, has been shown to play a critical role in maintaining glioblastoma, an aggressive adult brain malignancy, by epigenetically repressing expression of p53 target genes (57). This repression occurs through BRD8-dependent maintenance of H2A.Z occupancy at *p53* target loci, leading to a repressive chromatin state and facilitating the sustained proliferation of these cancer cells. The reason why H2A.Z functions as a repressor in this context remains unclear.

Recently, juxtaposed with another zinc finger 1 (JAZF1) was identified alongside malignant brain tumor domain containing 1 (MBTD1) as novel members of the EP400 complex, but not the SRCAP complex (58) (Fig. 1*B*). JAZF1 depletion results in a decreased level of H2A.Z acetylation (H2A.Zac) without affecting the nucleosome positioning of H2A.Z. These findings confirm the dual role of EP400 as both an H2A.Z depositor and a histone acetyltransferase (HAT) complex, an activity likely mediated by its unique TIP60 HAT component.

### Additional factors discovered to have H2A.Z-deposition potential

The SE translocation oncoprotein is homologous to the ATP-independent nucleosome assembly protein family, which functions as a histone chaperone (59). SE translocation has been shown to dynamically associate with the acidic C-terminal domain of H2A.Z following estrogen stimulation, promoting H2A.Z incorporation into chromatin at estrogen-responsive regulatory DNA elements (60) (Fig. 1*C*). This process establishes an active chromatin structure, facilitating gene expression in response to estrogen signaling.

Recently, the DNA-dependent protein kinase, which plays a crucial role in initiating DNA repair *via* the nonhomologous end-joining (NHEJ) pathway, has been identified as a putative (indirect) H2A.Z depositor (Fig. 1*C*) (61). DNA-dependent protein kinase phosphorylates H2A, resulting in a relaxation of the interaction between DNA and core histones. This process increases the accessibility of H2A.Z-H2B dimer, facilitating the exchange of H2A.Z for H2A.

A recent study by the group of Brendan Price raises the exciting possibility that H2A.Z exchange at sites of DNA damage might be facilitated by the nucleoside diphosphates linked to moiety-X (NUDIX) hydrolase NUDT5 (Fig. 1*C*) (62). NUTD5 hydrolyzes mono-ADP-ribose (mADPr) to ribose-5-phosphate and either AMP or ATP. The removal of NUDT5 results in decreased H2A.Z incorporation and reduced H4 acetylation at sites of DNA damage, suggesting that NUTD5 contributes (indirectly) to H2A.Z deposition during DNA repair. It remains to be determined whether this protein acts directly as H2A.Z depositor or indirectly by interacting with or modifying H2A.Z chaperone and remodeler complexes.

In addition to proteins, noncoding regulatory RNAs also appear to influence H2A.Z deposition. The long noncoding RNAs LIM homeobox 1 (LHX1)-DT and MIR503HG have been shown to affect H2A.Z chromatin recruitment (63, 64) (Fig. 1*C*), albeit through different mechanisms. LHX1-DT, which is expressed during early cardiomyocyte differentiation, indirectly associates with H2A.Z by binding to the H2A.Z interactor PHD finger protein 6. This interaction allows LHX1-DT to enrich H2A.Z at the LHX1 promoter region, thereby regulating gene expression needed for proper mesoderm differentiation into cardiomyocytes (63). The long noncoding RNA MIR503HG directly binds to H2A.Z, as identified through RNA pull-down assays followed by mass spectrometry and validated by immunoblots (64). MIR503HG is thought to negatively influence gene expression, as its overexpression affects H2A.Z recruitment to chromatin and results in increased H3K27me3 levels, likely by coordinating the deposition of H2A.Z in the promoter region of neurogenic locus notch homolog proteins 1 (NOTCH1).

### How to get H2A.Z out of chromatin: Meet the evictors INO80 and ANP32E

Not only is the faithful incorporation of H2A.Z into chromatin crucial for many cellular functions, its fine-tuned and precise eviction from target regions in exchange with H2A is also important. The most studied H2A.Z-specific evictors in mammals are INO80 (35) and ANP32E (33), which operate through different mechanisms and have been reported to possess additional, even controversial functions (Fig. 1*C*).

Research using proximal tubular rat cell lines supports INO80’s established role as a general H2A.Z evictor (65). Depletion of INO80 leads to an increased number of apoptotic cells, which correlates with an upregulated expression of proapoptotic genes, such as *PMAIP1*, whose loci exhibit elevated levels of (residual) H2A.Z. Here, H2A.Z functions as an activator of gene expression, while INO80 serves as specific evictor of this variant at genes that regulate apoptosis.

In addition to its role in apoptosis, INO80 appears to regulate the cell cycle in mammalian cells, and in this context, it seems to function as a depositor rather than an evictor of H2A.Z in certain cell types. A preprint study by the Richardson group demonstrate that INO80 depletion in oligodendrocytes precursors results in a slowed cell cycle characterized by a prolonged G1 phase, which correlates with increased H2A.Z mobility (66). This heightened mobility suggests that a significant portion of H2A.Z is freely diffusible and not chromatin-bound or incorporated, indicating that INO80 may act as a H2A.Z depositor in these particular cell types (Fig. 1*C*).

A similar depositor function of INO80 has also been identified in embryonic stem cells . INO80 plays a crucial role in controlling cellular differentiation by modulating the expression of developmental genes (67) (Fig. 1*C*). Notably, INO80 has minimal effects on self-renewal and transcriptional regulation in the naïve state. Surprisingly, contrary to its well-established role in H2A.Z exchange, INO80 enhances the occupancy of H2A.Z at bivalent promoters marked by H3K4me3 and H3K27me3 during the priming process.

The aforementioned studies reveal an unexpected dual role of INO80, indicating that it can function both as an H2A.Z evictor and as a depositor or chromatin stabilizer during early development.

In addition to INO80, ANP32E has also been identified as a specific evictor of H2A.Z (33, 34). A recent study by the group of Patrick Murphy supports this finding, demonstrating that the absence of Anp32e in mouse fibroblasts results in an additional accumulation of H2A.Z at promoters upstream of the transcriptional start site (68). The authors conclude that Anp32e acts to antagonize H2A.Z accumulation, thereby restricting chromatin accessibility genome-wide and subsequently changing chromatin states and gene expression levels.

Such a significant change in chromatin accessibility can be attributed to the induction and accumulation of R-loops at unstable genomic sites of transcription replication conflicts, as demonstrated in a recent preprint study (69). In patients with triple-negative breast cancer , ANP32E is frequently overexpressed due to MYC deregulation, resulting in a loss of H2A.Z at genomic sites characterized by increased R-loop length and a faster release of the initiating RNA polymerase II (69). Consequently, in triple-negative breast cancer cells, ANP32E functions as an evictor of H2A.Z, thereby regulating chromatin structure on a genome-wide scale.

In nondividing neurons, ANP32E seems to play a distinctly different role; it prevents the accumulation of H2A.Z under steady-state conditions rather than regulating the dynamics of H2A.Z in response to stimuli, such as KCl (70). Removal of H2A.Z in cultured hippocampal cells was not affected by the depletion of ANP32E, indicating that other factors are responsible for ejecting H2A.Z in these cells. Nevertheless, ANP32E remains essential for memory formation, transcription, and dendritic morphology, and these processes depend H2A.Z.

Further, a recent study by Dijkwel et al., has identified ANP32E as a molecular chaperone that regulates the protein stability of H2A.Z, thereby maintaining the soluble pool of this histone variant in the cytoplasm (71). Apparently, ANP32E interacts with H2A.Z in the G1 phase of the cell cycle in U2OS cells. ANP32E may act as a “buffer” in response to fluctuations in H2A.Z levels as they change throughout the cell cycle (Fig. 1*C*).

It remains unclear under what specific circumstances ANP32E and INO80 function as either evictors or stabilizers/depositors of H2A.Z. Additionally, it is not yet determined if these proteins exhibit a preference for one isoform of H2A.Z over the other, similar to what has been observed with the SRCAP complex (37). Understanding the context-dependent roles of these proteins and their potential isoform-specific interactions will be crucial for unraveling the complex regulatory mechanisms governing H2A.Z dynamics in various cellular environments.

In conclusion, although H2A.Z-specific depositors and evictors have been recognized and extensively studied for many years, recent research has revealed that these proteins exhibit diverse and sometimes opposing functions. This underscores the complexity of H2A.Z regulation and suggests the involvement of additional proteins that facilitate its dynamic “Ins and Outs” within the chromatin landscape. As our understanding of these mechanisms deepens, it opens new avenues for exploring the roles of H2A.Z in various biological processes and disease states.

## News on H2A.Z’s role in chromatin organization, gene regulation, and differentiation

When Dave discovered *hv1* in *Tetrahymena* in 1982 (7), he may not have anticipated that this finding would open the equivalent of a “Pandora’s box” of histone variants, revealing the extensive range of functions associated with H2A.Z. Since then, H2A.Z has been implicated in nearly all DNA-related processes including transcriptional activation and repression, DNA replication and repair (8, 10). Moreover, multiple studies have identified H2A.Z as a crucial factor for proper development, differentiation, and neuronal function (reviewed in (30, 72)). Thus, H2A.Z can be considered a versatile chromatin component, though its precise mechanistic roles remain often elusive: The more we learn, the more complex it becomes.

### A wild player: H2A.Z nucleosome dynamics and its implications for gene regulation

**Figure 2:**
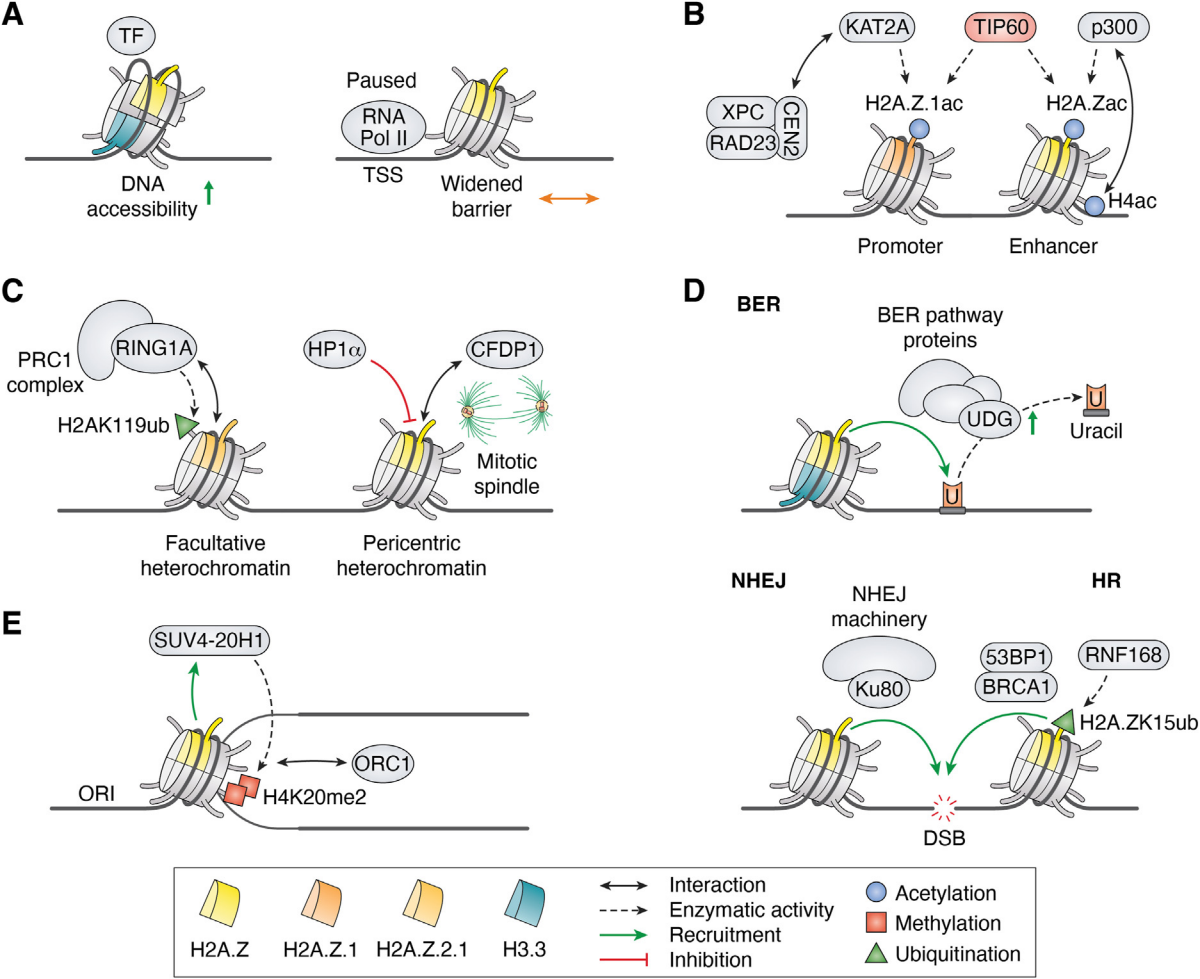
**H2A.Z regulates various cellular processes**. *A*, H2A.Z in nucleosome dynamics. H2A.Z incorporation influences nucleosome dynamics by increasing DNA accessibility and widening the nucleosomal barrier. This enhanced accessibility facilitates the binding of transcription factors (TF) and has implications for RNA-Pol II dynamics. *B*, novel findings on acetylation of H2A.Z at regulatory regions. At euchromatic regulatory regions, H2A.Z is acetylated by TIP60, p300, and KAT2A. KAT2A acetylation of H2A.Z.1 at a subset of promoters is licensed by NER pathway proteins XPC-RAD23-CEN2. p300 catalyzed acetylation at enhancers is increased by its direct interaction with H4 acetylation. *Blue circles* = acetylation. *C*, H2A.Z’s roles in condensed chromatin. In heterochromatin (HC), H2A.Z is both involved in facultative HC as well as pericentric HC establishment/maintenance. Within facultative HC, H2A.Z.2.1 recruits PRC1 and facilitates H2AK119ub through RING1A. At pericentric HC, H2A.Z occupancy is inhibited by HP1α. CFDP1 interaction is required for proper mitotic spindle formation. Green triangle = monoubiquitination. *D*, H2A.Z is involved in different DNA repair pathways. H2A.Z/H3.3 nucleosomes increase DNA accessibility for BER pathway proteins and enhance UDG activity to facilitate Uracil excision. At DSBs, H2A.Z recruits Ku80 for NHEJ, and HR pathway proteins through H2A.ZK15ub, which is mediated by RNF168. BER = base excision repair; HR = homologous repair; NHEJ = nonhomologous end joining. *Green triangle* = monoubiquitination. *E*, H2A.Z is implicated in DNA replication. H2A.Z licenses ORIs by recruitment of SUV4-20H1 and subsequent dimethylation of H4K20, leading to ORC1 binding. *Red squares* = methylation. CFDP1, craniofacial development protein 1; PRC1, polycomb repressive complex 1; UDG, uracil DNA glycosylase; ORC1, origin recognition complex subunit 1; ORI, origins of replication; KAT2A, lysine acetyltransferase 2A; NER, nucleotide excision repair.

In the past 5 years, research has focused extensively on the role of H2A.Z in nucleosome dynamics, particularly its functions in chromatin accessibility and gene regulation (Fig. 2*A*). For instance, Wen *et al.* analyzed nucleosome unwrapping states in mESCs and found that H2A.Z nucleosomes are enriched with unwrapping states (73). Especially the +1 nucleosomes of active genes appear to be more unwrapped compared to canonical H2A nucleosomes. As expected, depletion of H2A.Z results in decreased unwrapping of H3.3 nucleosomes, but surprisingly, it also leads to increased binding of CTCF (73). Additional evidence supporting these findings comes from another study demonstrating that the incorporation of H2A.Z lowers the energy barrier for DNA unwrapping, resulting in increased octamer plasticity and enhanced nucleosome gapping (74). This facilitates improved accessibility of DNA to various cellular processes. Unexpectedly, both N and C termini of H2A.Z are required for H2A.Z’s role in nucleosome dynamics. These observations are further supported by a structural model of H2A.Z in chromatin regulation (75), which suggests that H2A.Z incorporation increases DNA mobility due to its shorter C terminus compared to H2A. This also enables nucleosome arrays to form more condensed chromatin fibers, providing an additional explanation for H2A.Z’s dual role in transcription.

Moreover, other studies have demonstrated that H2A.Z-containing nucleosomes undergo spontaneous sliding on a sub-second timescale, a process that depends on H2A.Z’s N-terminus (76). H2A.Z also plays a role in nucleosome spacing and the regulation of repeat length, mediated by the SWI/SNF-related matrix-associated actin-dependent regulators of chromatin subfamily A member 5 (SMARCA5) (77). SMARCA5 colocalizes with CTCF at H2A.Z genomic sites, and SMARCA5 depletion leads to a loss of CTCF DNA binding and disruption of nucleosomal phasing at H2A.Z sites (77). Consistent with its role in chromatin architecture, H2A.Z incorporation at promoters is essential for maintaining an open chromatin structure (78). It achieves this by destabilizing nucleosomes and by increasing DNA accessibility for TFs. However, these functions are context dependent.

In addition to directly acting on the chromatin structure, H2A.Z.1 can also influence transcription by affecting RNA-polymerase II (RNA-Pol II) activity (Fig. 2*A*). A recent study demonstrates that H2A.Z plays a role in the pause-release mechanism of RNA-Pol ll (79). H2A.Z.1 slows the release of RNA-Pol ll from its paused state by modulating the dynamics of the negative elongation factor, thereby limiting the recruitment of the preinitiation complex in mESCs. The authors suggest that these findings align with H2A.Z’s essential role in embryonic development by coordinating rapid and synchronous gene expression. This notion is further supported by an independent *in vitro* study revealing that H2A.Z incorporation widens the nucleosome barrier and redistributes barrier strength (80). These nucleosomes occupy a larger region of DNA which influences DNA accessibility and RNA-Pol II dynamics.

H2A.Z’s genomic localization at regulatory regions, such as enhancers and promoters, is well established (20, 21) and its hyperacetylation has been shown to correlate strongly with gene activation (24). New findings provide additional context for the relationship between H2A.Z acetylation and the function of regulatory DNA regions (Fig. 2*B*). Two HATs, p300 and lysine acetyltransferase 2A (KAT2A, previously known as GCN5), have been identified as being responsible for acetylating H2A.Z at regulatory regions (81, 82). In addition to the already established function of TIP60, p300 has been shown to acetylate H2A.Z at enhancers (81). This acetylation is enhanced by H4ac, which significantly overlaps with H2A.Zac genome-wide, which interacts directly with the bromodomain of p300. It is proposed that different acetylation states of H2A.Z and H4 at enhancers define their activity. Supporting this, TIP60 does not efficiently acetylate H2A.Z *in vitro*; however, its genetic inhibition reduces acetylation in cellular systems (81). This finding suggests the existence of a regulatory network with multiple enzymes acting in concert depending on cellular contexts. KAT2A has been identified as a HAT that specifically acetylates H2A.Z.1 and not H2A.Z.2.1 at a subset of active promoters (82). Interestingly, the XPC-RAD23-CEN2 nucleotide excision repair complex functions as a coactivator, licensing the KAT2A-catalyzed acetylation of H2A.Z.1. This establishes a connection between DNA repair and transcriptional activity. *In vitro* experiments suggest that the isoform selectivity is attributable to the unique alanine at position 14 of H2A.Z.2.1, which inhibits the activity of KAT2A.

However, despite the substantial evidence supporting H2A.Z’s eminent role in transcription, another study suggests that H2A.Z is dispensable for both basal and activated transcription in postmitotic muscles (83). Considering this finding, H2A.Z’s function as transcriptional regulator may be most critical in cycling cells during differentiation and development.

### H2A.Z in chromatin organization

Besides studies of H2A.Z in active transcription and its localization in euchromatin, recent findings have also illuminated its role in transcriptionally silent heterochromatic regions (Fig. 2*C*). Previous research has established that H2A.Z is involved in centromere function (22) and monoubiquitylation of its C terminus has been found in heterochromatin (27, 28). Two recent publications now focus on the enigmatic function of H2A.Z in PCH (84, 85). In the first study, Gonzalez *et al.* examined a possible connection between H2A.Z and heterochromatin protein 1 (HP1) isoforms and discovered that within PCH, HP1α acts as a negative regulator of H2A.Z.1 (84). Specifically, depletion of HP1α, but not HP1β, leads to an accumulation of H2A.Z.1. Strikingly, the loss of H2A.Z.1 in PCH promotes hyperheterochromatinization by accumulating the epigenetic hallmarks H4K20me3, H3K27me3, and K3K9me3, resulting in increased genomic instability due to centromeric defects. Gopinathan *et al.* further connect H2A.Z and PCH to the regulation of mitotic spindle formation (85). Knockdown of the craniofacial development protein 1 results in a significant reduction of H2A.Z at pericentric repeat elements, reducing Ran GTPase activity due to diminished binding of regulator of chromosome condensation 1 and thereby interfering with the nucleation of microtubules. Rescue experiments involving a simultaneous KO of ANP32E partially restored RanGTP levels and alleviated the craniofacial defects associated with craniofacial development protein 1 loss, thereby connecting this H2A.Z evictor to mitotic functions.

Further support for H2A.Z’s role in heterochromatin comes from a preprint linking H2A.Z.2.1 to H2AK119ub and polycomb repressive complex 1 silencing (86). In this study, H2A.Z.2.1 is identified as a crucial component of H2AK119ub-marked nucleosomes and is likely to interact with RING1A *in vivo*.

Interestingly, H2A.Z has also been implicated in a novel type of silent chromatin, as evidenced by two different studies. Kafer *et al.* found that during trophoblast differentiation of hESCs, H2A.Zac is sequentially enriched at the nuclear periphery, followed by enrichment of H3K9me2 (87). They propose H2A.Zac may prime chromatin for subsequent silencing through H3K9me2. Similarly, Spracklin *et al.* identified a silent chromatin state enriched for H2A.Z and H3K9me2 in HCT116 cells (88). This state exhibits neutral three-dimensional interaction preferences in Hi-C, indicating that it neither interacts with nor excludes other compartments, which distinguishes it from other types of heterochromatin. However, they hypothesize that H2A.Z interferes with heterochromatin deposition and spreading, an effect that has already been demonstrated in yeast (89).

Recent reports suggest that H2A.Z.1 and H2A.Z.2.1 exhibit both parallel and antagonistic gene regulatory functions (19, 90). These isoforms can compensate for each other at certain promoters, yet they play opposing roles at a subset of other genes, with H2A.Z.1 functioning more as an activator and H2A.Z.2.1 acting more as a repressor (90). This different function may be attributed to specific preferences for binding partners, such as plant homeodomain finger protein 14 for H2A.Z.1 and the HDAC sirtuin-1 for H2A.Z.2.1, highlighting the importance of relative isoform levels in ensuring proper gene expression. Additionally, these isoforms may operate independently in controlling processes such as cell cycle progression and chromosome segregation (19). H2A.Z.1 appears to specifically regulate cell cycle genes, including Myc and Ki-67, during G1 phase progression, while H2A.Z.2.1 plays a role outside of transcription in maintaining centromere integrity and ensuring accurate chromosome segregation by facilitating proper spindle checkpoint function.

### Beyond gene regulation H2A.Z in DNA repair and replication

For many years, H2A.Z has been implicated in playing a role in double-strand break repair, where it first accumulates at the repair site and is subsequently evicted to promote further repair processes (23). In the past 5 years, additional reports have emerged on the function of H2A.Z in the different DNA repair pathways (base excision repair, NHEJ, and homologous recombination (HR)) (Fig. 2*D*) (91, 92, 93, 94).

In agreement with reports showing that H2A.Z deposition influences nucleosome dynamics (73, 74, 75, 76, 78), H2A.Z/H3.3-double variant nucleosomes have been demonstrated to enhance the excision of uracil by providing base excision repair pathway proteins with better access to the DNA lesion and increasing the activity of uracil DNA glycosylase (91, 92). These H2A.Z/H3.3-double variant nucleosomes exhibit a six-fold increase in cooccupancy compared to bulk nucleosomes (95).

The study by Belotti et al., investigated the longevity-related effects of H2A.Z depletion and demonstrated a striking accumulation of DNA damage, along with other premature aging effects in mouse skeletal muscle cells lacking H2A.Z (93). They further identified H2A.Z’s role in initiating double-strand break repair, where it directly recruits Ku80 through its vWA domain to the site of damage, thereby forming a molecular platform that is crucial for the NHEJ repair pathway.

In addition to the SUMOylation of H2A.Z.2, which has been shown to regulate its exchange at DNA damage sites (26), H2A.ZK15 ubiquitination by RING finger protein 168 has now been linked to the HR pathway (94). Specifically, the alpha1-extension helix of H2A.Z appears to be important for proper recruitment and orientation of RING finger protein 168, facilitating the setting of the ubiquitin modification that subsequently recruits different HR pathway factors to DNA damage sites.

It remains puzzling that H2A.Z seems to be both incorporated and evicted from DNA damage sites; therefore, further research is required to elucidate the connections between H2A.Z and the DNA damage repair.

H2A.Z appears to be also implicated in DNA replication (Fig. 2*E*). Although knowledge regarding the complex chromatin features of replication origins remains limited, experimental evidence has begun to connect H2A.Z to this process. New research demonstrates its enrichment at origins of replication (ORIs) (96, 97) and provides compelling evidence that H2A.Z serves as an important epigenetic hallmark of ORIs (98). Long *et al.* have shown that H2A.Z licenses ORIs by recruiting the methyltransferase SUV4-20H1, which subsequently dimethylates H4K20. This PTM is needed for the binding of the origin recognition complex subunit 1 and the subsequent activation of ORIs. Notably, these H2A.Z-regulated ORIs exhibit higher firing efficiency compared to other ORIs that lack H2A.Z, and depletion of H2A.Z results in decreased H4K20me2, origin recognition complex subunit 1 binding, and nascent DNA strands. The interaction between H2A.Z and SUV4-20H1 has been further characterized and structurally validated by cryo-EM studies (99, 100).

Thus, replication should be added to the growing list of processes in which H2A.Z is involved.

### H2A.Z in memory, differentiation, and development

Historically, H2A.Z has been recognized for its role in development and differentiation. For instance, H2A.ZK7me1 has been shown to be required for mESC self-renewal (25). Additionally, H2A.Z has been identified as a key regulator in neurodevelopment (29, 30), and in response to environmental changes (31).

Recent reports have further established connections between H2A.Z isoforms and development and differentiation across multiple cell types, highlighting their significant role in memory formation. In particular, the H2A.Z.2.1 isoform has been identified as a crucial regulator of microglial development (101). Deletion of H2A.Z.2.1 in neural progenitor cells results in an abnormal increase in microglial numbers, likely due to the aberrant expression of C-X-C motif chemokine ligand 14 (Cxcl14). H2A.Z.2.1 recruits the H3K9 methyltransferase G9a to the promoter of the *Cxcl14* gene, thereby repressing its expression. Beyond its role in microglial development, H2A.Z.2.1 appears to be also important for the differentiation of neural crest cell into melanocytes (102). In this model system, H2A.Z.2.1 regulates the melanocyte-inducing transcription factor (Mitf), a key regulator of melanocyte development, by occupying its promoter and enhancing its induction. Silencing of H2A.Z.2.1 leads to a significant reduction in the number of melanocyte precursors in zebrafish and mESCs.

Another study found that H2A.Z contributes to neuronal survival by controlling the expression of nuclear-encoded mitochondrial genes (103). Additionally, our research has demonstrated that the H2A.Z-associated high-mobility group 20 A (HMG20A) protein plays a crucial role in early head and heart differentiation. HMG20A is located at nucleosome depleted regions of promoters that are surrounded by H2A.Z nucleosomes and bound by PWWP domain-containing protein 2A (PWWP2A) (104). Moreover, Cameron *et al.* reported that the loss of the elongator subunit Elp1 results in the increased abundance of H2A.Z in chromatin, while simultaneously reducing the acetylation of H2A.Z (105). These changes are associated with perturbations in the Notch signaling pathway and neuronal development. Treatment with the HDAC inhibitor trichostatin A rescues the development of sensory neurons by correcting Notch3 expression patterns. Furthermore, H2A.Z.1 acetylation is essential for proper memory formation (106).

The group led by Rispal et al., investigated the role of H2A.Z in intestinal epithelial homeostasis and found that H2A.Z integrates Wnt signaling to regulate proliferation, differentiation marker expression and the recruitment of key TFs such as caudal type homeobox 2 (CDX2) (107). Through these mechanisms, H2A.Z ensures the proper maintenance of the intestinal epithelium. A recent preprint from the same group further explores the functional differences between the H2A.Z isoforms in intestinal homeostasis (108). Both isoforms appear to be enriched in gene bodies during enterocyte differentiation; however, H2A.Z.1 represses terminal differentiation, whereas H2A.Z.2.1 promotes it. Conversely, both isoforms cooperate to facilitate proliferation and differentiation of stem and progenitor cells into secretory lineages. This underscores the notion that H2A.Z isoforms possess both redundant and specific functions in gene regulation.

H2A.Z appears to also play a role in the maintenance of HSCs (109, 110). Numata *et al.* revealed the critical importance of TIP60 and H2A.Zac in HSC function (109). Loss of TIP60 results in a significant reduction of H2A.Zac and impaired HSC functionality, likely due to disrupted transcription of Myc target genes. Further, it has been demonstrated that specific HSC-specific enhancers are sensitive to stimulation by prostaglandin E2 (PGE2) and 16,16-dimethyl-PGE2 (dmPGE2), which lead to the incorporation and acetylation of H2A.Z, induction of acute inflammatory gene and promotion of HSC fate (110).

## H2A.Z “gone bad”: how its deregulation contributes to diseases

In 2016, Dave et al., alongside numerous other research groups, coined the term "oncohistones" to describe cancer-driving histone mutations (111, 112, 113). These mutations, which had been independently identified by various groups the years prior (114, 115, 116, 117, 118, 119, 120), typically involve exchanges of lysine for methionine within histone H3 genes, such as H3.3K27M (121) or H3.3K36M (122).

This intriguing concept raises the question whether H2A.Z also contributes to disease development and whether it could function as an oncohistone? Given that the essential histone variant H2A.Z is a key regulator of chromatin function and plays a crucial role in mammalian development, its deregulation or mutation could have profound effects on cell fate and potentially lead to diseases.

### Too much is never good: H2A.Z the “bad guy” in multiple cancers

**Figure 3:**
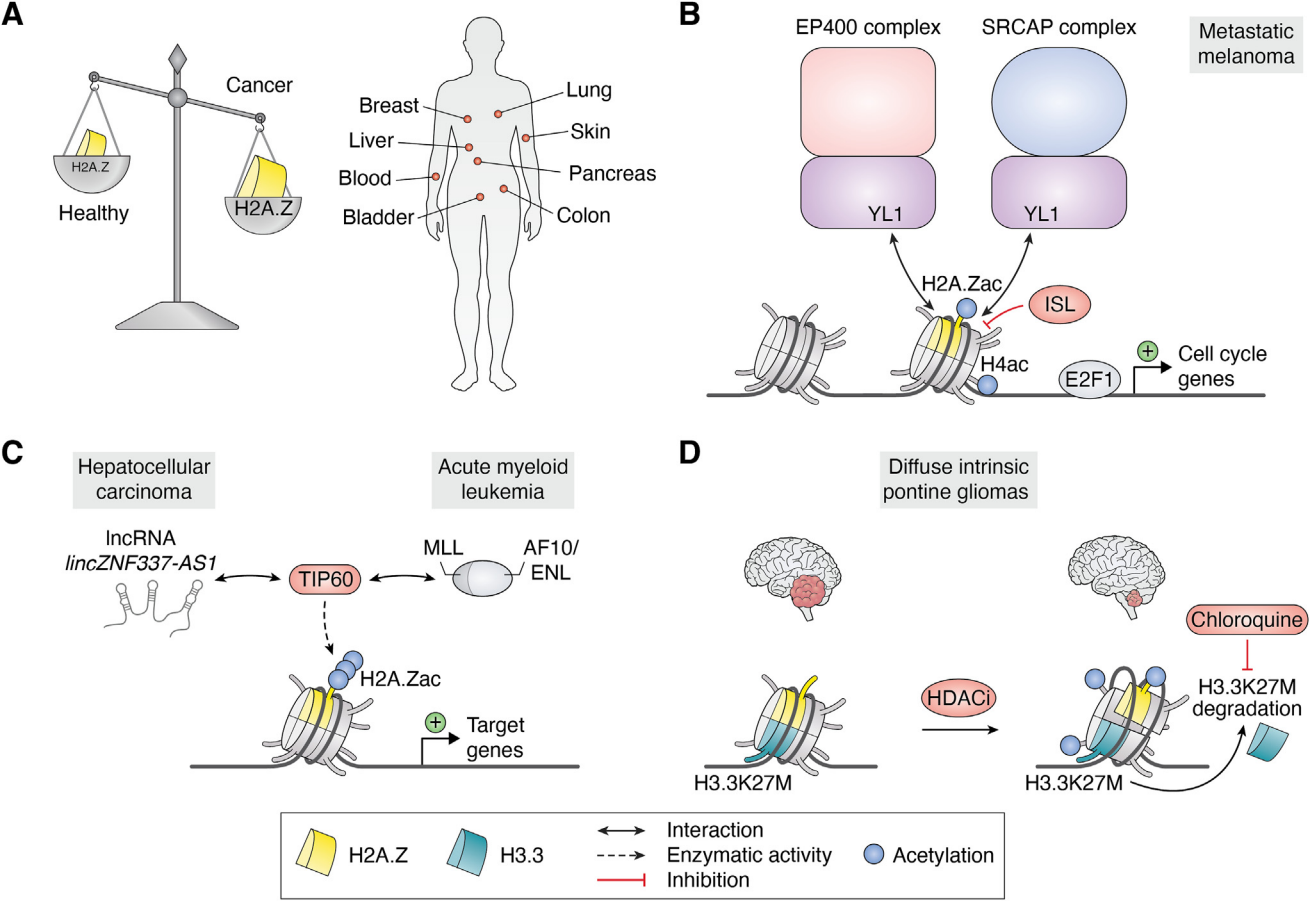
**Deregulation of H2A.Z in cancers**. *A*, overexpression of both H2A.Z isoforms is a hallmark of multiple cancers. Depicted are known cancers with deregulated H2A.Z levels. *B*, H2A.Z promotes metastatic melanoma. In metastatic melanoma, EP400 and SRCAP complexes, along with their shared subunit YL1, contribute to tumorigenesis by incorporating H2A.Z near E2F1 target genes and by stabilizing acetylation levels of H4 and H2A.Z. Isoliquiritigenin (ISL) has the potential to serve as a therapeutic agent, as it downregulates H2A.Z expression. Gene activation is represented by *green* “+” *circle*. *Blue circles* = acetylation. *C*, hyperacetylated H2A.Z contributes to tumorigenesis. In both HCC and AML patients, TIP60 is recruited to H2A.Z sites by specific lncRNA or MLL fusion proteins, leading to hyperacetylated H2A.Z-containing nucleosomes and an upregulation of oncogenic target genes. Gene activation is represented by *green* “+” circle. *Blue circles* = acetylation. *D*, H2A.Z supports HDAC inhibitors in the treatment of a certain brain tumor. In DIPG, the presence of H2A.Z is beneficial for cells treated with HDAC inhibitors (HDACi). H2A.Z likely destabilizes nucleosomes containing the oncohistone H3.3K27M through hyperacetylation, facilitating the displacement and degradation of this oncohistone. This process is inhibited by chloroquine treatment. *Blue circles* = acetylation. DIPG, diffuse intrinsic pontine glioma; E2F1, E2 promoter binding factor 1; AML, acute myeloid leukemia; HDAC, histone deacetylase; SRCAP, snf2-related CREBBP; HCC, hepatocellular carcinoma cell.

Indeed, H2A.Z can function as an oncogene, as its overexpression has been detected in various tumors, including breast, colon, liver, lung, and bladder cancers, as well as metastatic melanoma and pancreatic ductal adenocarcinomas (PDACs) (reviewed in (8, 123)) (Fig. 3*A*).

Strikingly, distinct mechanistic functions of the two isoforms H2A.Z.1 and H2A.Z.2.1 were first discovered in malignant melanoma (124). In this aggressive cancer, H2A.Z.2.1 promotes cell proliferation by regulating the expression of E2 promoter binding factor 1 (E2F1) target genes. It stabilizes the acetylation of both H3 and H4 in the chromatin surrounding E2F1-regulated promoters, enhancing the binding of BRD2 and E2F1, which in turn upregulate genes that drive cell cycle progression. Depletion of H2A.Z.2.1 induces cell cycle arrest and sensitizes cells to therapy with inhibitors targeting the bromodomain and extraterminal domain and mitogen-activated protein kinase kinase. A recent follow-up study now provides deeper insights into H2A.Z’s role in melanoma, particularly regarding the chaperone complexes that facilitate H2A.Z incorporation into chromatin (125). In melanoma, both the H2A.Z-specific SRCAP and EP400 chaperone complexes bind directly to E2F1 target genes (Fig. 3*B*). By individually depleting *SRCAP, p400*, and *YL1* genes the authors show that not only H2A.Z deposition into chromatin is severely affected, but that also H4 acetylation is lost at promotors of cell cycle genes, which are direct H2A.Z targets. The common YL1 subunit of both complexes is particularly important; its overexpression correlates with poor prognosis, and a knockdown of YL1 induces apoptosis in melanoma cells. Thus, H2A.Z and its chaperones are evidently “bad guys” in the context of melanomas.

Identifying a substance or treatment that reduces H2A.Z expression could significantly extend lifespan or improve therapeutic outcomes when combined with bromodomain and extraterminal domain and mitogen-activated protein kinase kinase inhibitors. Recently, isoliquiritigenin (2′,4′,4-trihydroxychalcone, ISL), a naturally occurring compound extracted from the Chinese liquorice plant (*Glycyrrhiza uralensis*), has emerged as a promising candidate (reviewed in (126)). Previously reported for its antitumor properties, ISL may also be effective in reducing H2A.Z expression, potentially enhancing therapeutic efficacy in melanoma. Xiang et al., investigated the effects of ISL on melanoma cells, finding that both isoforms of H2A.Z are significantly downregulated in a dose-dependent manner following ISL administration (127). Furthermore, ISL inhibits epithelial-mesenchymal transition and induces cell cycle arrest, resulting in a substantial reduction in melanoma cell proliferation. These effects are attributed to the ISL-dependent downregulation of H2A.Z, as the overexpression of H2A.Z in ISL-treated cells reverses the observed phenotypes (Fig. 3*B*).

Several publications have examined the relationship between H2A.Z and liver cancer. For instance, Dong et al., focused on H2A.Z.1 and found that its upregulation in hepatocellular carcinoma, compared to normal tissue, correlates with poor prognosis (128). By analyzing data from the Cancer Genome Atlas Liver Hepatocellular Carcinoma collection, the researchers identified a correlation between H2A.Z.1 overexpression and mutations in the tumor protein p53 (TP53). Similar to findings in melanomas, H2A.Z.1 overexpression in hepatocellular carcinoma (HCC) cells is associated with increased cell proliferation, migration, and invasion. Liver cancer patients exhibiting high H2A.Z.1 expression and TP53 mutations have the worst prognosis and highest risk of tumor progression, while those with low H2A.Z.1 expression and wild-type TP53 show the best prognosis. Consequently, the authors propose utilizing H2A.Z.1 expression as a prognostic biomarker for this specific type of cancer.

Tang *et al.* also examined the differences between H2A.Z.1 and H2A.Z.2.1 in liver cancer by primarily using data mining methods (129). Similar to the findings of Dong *et al.*, they observed overexpression of both H2A.Z isoforms in hepatocellular carcinoma. These elevated levels correlate with advanced cancer stages and a poor prognosis. The authors analyzed coexpressed genes of either overexpressed H2A.Z.1 or H2A.Z.2.1 in hepatocellular carcinomas and identified distinct pathways associated with each isoform. H2A.Z.1 was found to be coexpressed with genes involved in splicing and RNA transport. In contrast, H2A.Z.2.1 was shown to be coexpressed with genes encoding proteins localized to the kinetochore, spindle midzone, and microtubule binding. The authors conclude that while both H2A.Z isoforms play similar roles in cell proliferation, their oncogenic mechanisms appear to be distinct in hepatocarcinogenesis.

In a study investigating the role of H2A.Z in radiation resistance in lung adenocarcinomas was examined (130). The authors found that H2A.Z.1 is frequently overexpressed in lung adenocarcinomas, indicating a positive association with disease progression and poor prognosis. Silencing H2A.Z.1 inhibits migration, invasion, and epithelial-mesenchymal transition in lung cancer cell lines, and it also contributes to radiation resistance. Notably, depleting H2A.Z.1 sensitizes lung cancer cells to radiation treatment.

H2A.Z also has a detrimental role in PDAC, a tumor known for its challenging treatment and thus classified as one of the most intractable and devastating malignant cancers. PDAC exhibits resistance to gemcitabine, the standard first-line chemotherapy. Avila-Lopez et al., investigated the role of H2A.Z in PDAC, specifically focussing on its function in chemotherapy resistance (131). All isoforms of H2A.Z are overexpressed in PDAC. Depletion of H2A.Z induces G2/M phase cell cycle arrest, indicating its proliferative effect in PDAC, which is further supported by the observed reduction in tumor growth following H2A.Z depletion *in vivo*. Additionally, H2A.Z knockdown alters the expression of genes involved in senescence, resulting in increased senescence. Notably, reduced H2A.Z expression also reverses chemo-resistance, positioning H2A.Z as a potential prognostic marker and a promising pharmacological target for this otherwise challenging tumor type in the future.

Researchers from the same institute have also explored the oncogenic role of H2A.Z in cervical cancer (132). They observed overexpression of H2A.Z in this cancer type as well, with their study focusing on the molecular mechanisms underlying this overexpression. Promoter studies revealed that H2A.Z overexpression is influenced by DNA methylation, copy number variations, and the TFs AP2α and ETS-like-1 (ELK1). Additionally, H2A.Z itself binds to promoters and enhancers of genes involved in cancer-related pathways. In these regions, H2A.Z facilitates the recruitment of TFssuch as nuclear respiratory factor 1, nuclear transcription factor Y subunit alpha, as well as RNA-Pol II, underscoring its potential as a therapeutic target.

### H2A.Z and its PTMs in cancer: Small chemical changes and their deleterious outcomes

Not only does the overexpression of H2A.Z impact the fate of cancer cells, but its various PTMs also play a significant role. A recent study explored the implications of H2A.Z acetylation in HCC (133). Consistent with the previously cited studies, the researchers found that H2A.Z promotes the proliferation of HCC cells, reduces their apoptosis, and facilitates invasion and metastasis. Furthermore, H2A.Z is more highly acetylated in HCC tissues compared to normal tissues. Through an RNA immunoprecipitation screen, the researchers identified a long intergenic noncoding RNA, lincZNF337-AS1, which binds to both acetylated and nonacetylated H2A.Z. High levels of lincZNF337-AS1 correlate with poor prognosis in HCC patients. Surprisingly, lincZNF337-AS1 enhances the acetylation of H2A.Z (Fig. 3*C*). KO of lincZNF337-AS1 reduces H2A.Z acetylation and alters the expression of all tested downstream target genes of H2A.Z. The HAT responsible for this acetylation is TIP60, a component of the EP400 H2A.Z chaperone complex. In summary, TIP60/lincZNF337-AS1-mediated acetylation of H2A.Z promotes downstream target gene expression and contributes to the oncogenic potential of H2A.Z.

Another example of H2A.Z acetylation playing a role in carcinogenesis is found in acute myeloid leukemia. The molecular basis of acute myeloid leukemia involves the translocation of the mixed-lineage leukemia (MLL) gene, which leads to the production of oncogenic fusion proteins. These proteins activate the transcription of genes from the homeobox A (HoxA) cluster and myeloid ecotropic viral integration site 1. TIP60 is recruited to the *Hoxa9* locus through its interaction with two fusion proteins, MLL-AF10 and MLL-ENL, where it acetylates H2A.Z (134) (Fig. 3*C*). Transplantation studies using TIP60 knockdown cells have demonstrated that TIP60-mediated acetylation of H2A.Z is essential for leukemogenesis driven by MLL-AF10 and MLL-ENL *in vivo*.

Another modification of H2A.Z related to cancer was identified during efforts to find novel strategies to sensitize colorectal cancer to radiotherapy. This is especially pertinent, as resistance to radiotherapy is a significant challenge in the treatment of colon cancer. Through content screening and analysis of tissue arrays from colorectal cancer patients who are either resistant or sensitive to radiotherapy, the study identified SENP5, a member of the sentrin-specific protease (SENP) family known for its deSUMOylation activity (135). Patients with high SENP5 expression exhibited increased resistance to radiotherapy. Through SUMO mass spectrometry analysis, it was found that the main target of SENP5 deSUMOylation is H2A.Z, which is SUMOylated at three lysine residues. This discovery is particularly important as the E3 ligase responsible for H2A.Z SUMOylation had yet to be identified. SENP5 enhances HR-mediated DNA repair by deSUMOylating H2A.Z at these three lysine sites, which subsequently influences the recruitment of downstream HR factors. The authors suggest that SENP5, as a deSUMOylase of H2A.Z, could serve as a potent prognostic marker and intervention target for cancer radiotherapy.

### H2A.Z and brain defects

Beyond its role in tumors, H2A.Z also plays critical roles in brain development and function. Gretzinger et al., investigated gene expression differences in the hippocampus of rats whose mothers were exposed to alcohol, modeling human fetal alcohol spectrum disorder (FASD) (136). This disease is caused by prenatal ethanol exposure and is characterized by lifelong physical and cognitive deficits, including impairments in executive functioning, social skills, and, notably, learning and memory. The authors focused on methyl CpG binding protein 2 (MeCP2) and H2A.Z in this animal model of FASD. They found that while methyl CpG binding protein 2levels in the brain remain unchanged between ethanol-treated and control rats, H2A.Z is downregulated in the hippocampus of rats exposed to ethanol during the prenatal phase. More specifically, they observed that H2A.Z.2.1 is strongly downregulated, whereas H2A.Z.1 remains relatively unchanged, suggesting that only the H2A.Z.2.1 isoform plays a role in FASD. This finding once again implies the existence of distinct H2A.Z isoform-specific functions, yet it does not solve the mystery behind the underlying mechanism. It raises important questions of how *H2AFZ* and *H2AFV* genes are differentially transcribed and how their gene products regulate distinct gene expression programs in different organs.

In a separate study, researchers investigated the role of H2A.Z in posttraumatic stress disorder (PTSD), contributing to ongoing research on H2A.Z’s involvement in brain functions (137, 138). PTSD develops in response to traumatic experiences and is characterized by intrusive memories of the trauma and heightened sensitivity to fear (138, 139). In addition, rates of chronic pain are notably higher among patients with PTSD compared to the general population. Studies involving female mice have shown increased binding of H2A.Z to all tested promoters in hippocampal cells compared to males (138). Conditional KO of both H2A.Z isoforms in specific neurons resulted in the downregulation of all tested genes. Intriguingly, these studies revealed that H2A.Z has sex-specific effects on fear memory: in males, H2A.Z regulates acute fear learning without affecting stress-mediated fear sensitization, whereas the opposite is observed in females. Furthermore, H2A.Z and stress-mediated fear sensitization contribute to the development of pain hypersensitivity, a prevalent issue in PTSD patients. Once again, H2A.Z surprises us—how can such a ubiquitous small nucleosomal component exert such significant influence on sex-specific memory effects?

### H2A.Z the “good guy”: Counteracting the oncohistones

In most diseases where H2A.Z is involved, its overexpression or improper modification tends to be detrimental, often exacerbating the condition. However, there is a notable exception where H2A.Z plays a beneficial role. In pediatric high-grade glioma cells, the presence of H2A.Z has been found to be advantageous (140). Diffuse intrinsic pontine gliomas (DIPGs) are a deadly and nonresectable type of brain tumor located in the brainstem. Currently, there are no effective therapies available, resulting in a poor prognosis and early patient mortality. The molecular basis of DIPGs involves mutations in histones H3.1 or H3.3 at position 27, where a lysine is replaced by a methionine (H3K27M). This mutation leads to a global loss of H3K27me3, likely due to the sequestering the polycomb repressive complex 2 member enhancer of zeste homolog 2, which strongly binds to the oncohistone H3K27M (121).

A recent study demonstrates that HDAC inhibitors effectively reduce levels of H3.3K27M oncohistone in primary DIPG cell lines derived from patients (140). This reduction, observable at the chromatin incorporation level, is associated with decreased cell viability, reduced proliferation, and increased apoptosis. Interestingly, H2A.Z enhances the effects of H3K27M displacement by HDAC inhibitors. The authors suggest that the cooccurrence of both H3.3K27M and H2A.Z at actively transcribed genes may facilitate the loss of H3K27M, particularly during increased histone acetylation induced by HDAC inhibition (Fig. 3*D*).

Thus, H2A.Z appears to also play a significant role in Dave’s oncohistone concept, acting as the "good guy" that counteracts the effects of other histones "gone bad."

In conclusion, much more research is needed to elucidate the mechanisms behind H2A.Z deposition or eviction at specific chromatin sites throughout various stages of the cell cycle. Additionally, it is crucial to explore how H2A.Z influences chromatin structure, gene transcription, replication, DNA repair, as well as the reasons behind its deregulation leading to different diseases. The presence of highly similar H2A.Z isoforms adds further complexity, as they exhibit either overlapping or unique functions depending on the context. As Dave aptly stated “Every amino acid matters”. He could not have been more right!

## Conflict of interest

The authors declare that they have no conflict of interest with the contents of this article.
